# Supplementary material for: Pharmacokinetic Evaluation of a Novel Transdermal Ketoprofen Formulation in Healthy Dogs
Source: Pharmaceutics. 2022 Mar 15;14(3):646. doi: 10.3390/pharmaceutics14030646 (PMC8953954; doi:10.3390/pharmaceutics14030646)
Supplement: Supplementary file 1 [file pharmaceutics-14-00646-s001.zip › pharmaceutics-1633709-supplementary.pdf]

pharmaceutics

Supplementary Materials: Pharmacokinetic Evaluation of a Novel Transdermal Ketoprofen Formulation in Healthy Dogs

Halley Gora Ravuri, Nana Satake, Alexandra Balmanno, Jazmine Skinner, Samantha Kempster and Paul C. Mills

Table S1. List of vehicles tested for solubility for meloxicam, tolfenamic acid, ketoprofen and carprofen.

Sl.no	Name of the vehicle	Drug conc at 1%				Drug conc at 5%			
		Meloxicam	Tolfenamic acid	Ketoprofen	Carprofen	Meloxicam	Tolfenamic acid	Ketoprofen	Carprofen
1.	Propylene glycol (PG)	dissolved	dissolved	dissolved	dissolved	precipitated and undissolved	precipitated and undissolved	dissolved#	precipitated and undissolved
2.	Isopropyl myristate (IPM)	dissolved	dissolved	dissolved	dissolved	precipitated and undissolved	precipitated and undissolved	dissolved#	precipitated and undissolved
3.	diethylene glycol monoethyl ether (DGME; Transcutol)	dissolved	dissolved	dissolved	dissolved	precipitated and undissolved	precipitated and undissolved	dissolved#	dissolved*
4.	Dimethyl sulphoxide (DMSO)	dissolved	dissolved	dissolved	dissolved	dissolved	precipitated and undissolved	dissolved#	dissolved*
5.	Stearyl octanoate and cetyl octanoate (SCO -LQ-(MH)	dissolved	dissolved	dissolved	dissolved	precipitated and undissolved	precipitated and undissolved	dissolved#	precipitated and undissolved
6.	Ethanol + Isopropyl myristate (50:50)	dissolved	dissolved	dissolved	dissolved	precipitated and undissolved	precipitated and undissolved	dissolved#	precipitated and undissolved
7.	Oleic acid (OA)	dissolved	precipitated	dissolved	dissolved	precipitated and undissolved	precipitated and undissolved	dissolved	precipitated and undissolved
8.	Oleic acid + Isopropyl myristate (50:50)	dissolved	dissolved	dissolved	dissolved	precipitated and undissolved	precipitated and undissolved	dissolved#	precipitated and undissolved
9.	Eucalyptus oil + (Isopropyl myristate – ethanol)	dissolved	dissolved	dissolved	dissolved	precipitated and undissolved	precipitated and undissolved	dissolved#	precipitated and undissolved
10.	Dimethyl formamide (DMF)	dissolved	dissolved	dissolved	dissolved	dissolved	precipitated and undissolved	dissolved#	dissolved*
11.	50% Dimethyl formamide in H20	dissolved	dissolved	dissolved	dissolved	precipitated and undissolved	precipitated and undissolved	dissolved#	dissolved*
12.	100% ethanol	dissolved	dissolved	dissolved	dissolved	precipitated and undissolved	precipitated and undissolved	dissolved#	precipitated and undissolved
13.	100% isopropanol	dissolved	dissolved	dissolved	dissolved	precipitated and undissolved	precipitated and undissolved	dissolved#	precipitated and undissolved

14.	50% ethanol in H2O	dissolved	precipitated	dissolved	dissolved	precipitated and undissolved	precipitated and undissolved	dissolved#	precipitated and undissolved
15.	50% isopropanol in H2O	dissolved	precipitated	dissolved	dissolved	precipitated and undissolved	precipitated and undissolved	dissolved#	precipitated and undissolved
16.	Dimethyl acetamide (DMA)	dissolved	dissolved	dissolved	dissolved	dissolved	precipitated and undissolved	dissolved#	precipitated and undissolved
17.	Benzyl alcohol	dissolved	dissolved	dissolved	dissolved	dissolved	precipitated and undissolved	dissolved#	dissolved*
18.	corn oil (CO)	dissolved	precipitated	dissolved	dissolved	precipitated and undissolved	precipitated and undissolved	dissolved	precipitated and undissolved
19.	Eucalyptus oil (EU)	dissolved	dissolved	dissolved	dissolved	precipitated and undissolved	precipitated and undissolved	dissolved#	precipitated and undissolved
20.	Limonene	dissolved	dissolved	dissolved	dissolved	precipitated and undissolved	precipitated and undissolved	dissolved#	precipitated and undissolved
21.	Eucalyptol (1,4-Cineole)	dissolved	dissolved	dissolved	dissolved	precipitated and undissolved	precipitated and undissolved	dissolved#	precipitated and undissolved
22.	50% DMSO in H2O	dissolved	dissolved	dissolved	dissolved	precipitated and undissolved	precipitated and undissolved	dissolved#	dissolved*
23.	Tween 80 (polysorbate 80)	dissolved	precipitated	dissolved	dissolved	precipitated and undissolved	precipitated and undissolved	dissolved#	precipitated and undissolved
24.	Span 20 (Sorbitan monolaurate)	dissolved	precipitated	dissolved	dissolved	precipitated and undissolved	precipitated and undissolved	dissolved#	precipitated and undissolved
25.	Methanol	dissolved	dissolved	dissolved	dissolved	precipitated and undissolved	precipitated and undissolved	dissolved#	dissolved*
26.	50% methanol + 50% Propylene glycol	dissolved	dissolved	dissolved	dissolved	precipitated and undissolved	precipitated and undissolved	dissolved#	precipitated and undissolved
27.	50% Propylene glycol + 50 % ethanol	dissolved	dissolved	dissolved	dissolved	precipitated and undissolved	precipitated and undissolved	dissolved#	precipitated and undissolved
28.	Stearyl octanoate + (Isopropyl myristate – ethanol)	dissolved	dissolved	dissolved	dissolved	precipitated and undissolved	precipitated and undissolved	dissolved#	precipitated and undissolved
29.	Dimethyl formamide + (Isopropyl myristate – ethanol)	dissolved	dissolved	dissolved	dissolved	precipitated and undissolved	precipitated and undissolved	dissolved#	dissolved*
30.	Dimethyl acetamide + (Isopropyl	dissolved	dissolved	dissolved	dissolved	precipitated and undissolved	precipitated and undissolved	dissolved#	dissolved*

	myristate – ethanol)								
31.	Eucalyptus oil + (Isopropyl myristate – isopropanol)	dissolved	dissolved	dissolved	dissolved	precipitated and undissolved	precipitated and undissolved	dissolved#	precipitated and undissolved
32.	Benzyl alcohol + (Isopropyl myristate – ethanol)	dissolved	dissolved	dissolved	dissolved	precipitated and undissolved	precipitated and undissolved	dissolved#	precipitated and undissolved
33.	Benzyl alcohol + (Isopropyl myristate – isopropanol)	dissolved	dissolved	dissolved	dissolved	precipitated and undissolved	precipitated and undissolved	dissolved#	precipitated and undissolved
34.	Isopropanol (45%), isopropyl myristate (45%)	dissolved	dissolved	dissolved	dissolved	precipitated and undissolved	precipitated and undissolved	dissolved#	precipitated and undissolved
35.	Isopropanol (45%), Transcutol (45%)	dissolved	dissolved	dissolved	dissolved	precipitated and undissolved	precipitated and undissolved	dissolved#	precipitated and undissolved
36.	5% menthol in Ethanol-H2O	dissolved	dissolved	dissolved	dissolved	precipitated and undissolved	precipitated and undissolved	dissolved#	precipitated and undissolved

*: Carprofen dissolved at 10% (*w*/*v*) concentration; #: Ketoprofen dissolved at 10% (*w*/*v*) concentration.

Table S2. List of vehicles tested for initial in vitro permeability of ketoprofen (10% *w*/*v*) through dog skin, to pick best penetration enhancers in final combinations.

Sl.no	Vehicles tested
1.	Ethanol (10%, 20%, 30%, 40%, 50%) in water
2.	Isopropanol (10%, 20%, 30%, 40%, 50%) in water
3.	Eucalyptus oil (1%, 5%, 10%) in isopropanol
4.	Menthol (1%, 5%, 10%) in water
5.	Limonene (1%, 5%, 10%) in isopropanol
6.	Isopropyl myristate (10%, 20%, 30%, 40%, 50%) in water
7.	Transcutol (10%, 20%, 30%, 40%, 50%) in water
8.	Oleic acid + Isopropyl myristate (50:50)
9.	Ethanol + Isopropyl myristate (50:50)
10.	Isopropanol + Isopropyl myristate (50:50)

Table S3. Final vehicle combinations tested for ketoprofen at 10% (W/V) concentration using Franz cells.

List of formulations	Combination of vehicles used for each formulation
F1	Ethanol (45%), oleic acid (45%), eucalyptus oil (10%)
F2	Ethanol (35%) isopropyl myristate (35%), Transcutol (20%) eucalyptus oil (10%).
F3	Ethanol (45%), Transcutol (45%), eucalyptus oil (10%)
F4	Isopropanol (45%), Transcutol (45%), eucalyptus oil (10%)
F5	Isopropanol (45%), isopropyl myristate (45%), eucalyptus oil (10%)
F6	Ethanol (45%), isopropyl myristate (45%), eucalyptus oil (10%)
F7	Ethanol (45%), Transcutol (45%), menthol (10%)
F8	Isopropanol (45%), Stearyl octanoate (45%), eucalyptol (10%)
F9	Isopropanol (45%), STSCQ (45%), eucalyptol (10%)
F10	Isopropanol (45%), PMP (45%), eucalyptus oil (10%)
F11	Isopropanol (45%), Polyethylene glycol 400 (45%), eucalyptus oil (10%)

F12	Ethanol (45%), polyethylene glycol 400 (45%), eucalyptus oil (10%)
F13	Ethanol (45%), isopropyl myristate (45%), limonene (10%)
F14	Ethanol (45%), isopropyl myristate (45%), menthol (10%)
F15	Ethanol (50%), isopropyl myristate (35%), *dimethylformamide (5%), eucalyptus oil (10%)*
F16	Ethanol (40%), isopropyl myristate (20%), eucalyptus oil (10%), transcutol (10%), *dimethylformamide (20%)*
